# Metagenomic surveillance and comparative genomic analysis of *Chlamydia psittaci* in patients with pneumonia

**DOI:** 10.3389/fmicb.2023.1157888

**Published:** 2023-05-30

**Authors:** Weifeng Huang, Shuqin Hu, Yongzhe Zhu, Shijia Liu, Xingya Zhou, Yuan Fang, Yihan Lu, Ruilan Wang

**Affiliations:** ^1^Department of Intensive Care Medicine, Shanghai Sixth People’s Hospital Affiliated to Shanghai Jiao Tong University School of Medicine, Shanghai, China; ^2^Department of Critical Care Medicine, Shanghai General Hospital of Nanjing Medical University, Shanghai, China; ^3^Department of Microbiology, Navy Medical University, Shanghai, China; ^4^Department of Pulmonary Disease, PLA 905 Hospital, Shanghai, China; ^5^Genoxor Medical Science and Technology Inc., Shanghai, China; ^6^Department of Epidemiology, Ministry of Education Key Laboratory of Public Health Safety, School of Public Health, Fudan University, Shanghai, China; ^7^Shanghai Institute of Infectious Disease and Biosecurity, Shanghai, China

**Keywords:** metagenomics, comparative genomics, *Chlamydia psittaci*, obligate intracellular pathogen, phylogeny, pangenome, positive selection

## Abstract

*Chlamydia psittaci*, a strictly intracellular bacterium, is an underestimated etiologic agent leading to infections in a broad range of animals and mild illness or pneumonia in humans. In this study, the metagenomes of bronchoalveolar lavage fluids from the patients with pneumonia were sequenced and highly abundant *C. psittaci* was found. The target-enriched metagenomic reads were recruited to reconstruct draft genomes with more than 99% completeness. Two *C. psittaci* strains from novel sequence types were detected and these were closely related to the animal-borne isolates derived from the lineages of ST43 and ST28, indicating the zoonotic transmissions of *C. psittaci* would benefit its prevalence worldwide. Comparative genomic analysis combined with public isolate genomes revealed that the pan-genome of *C. psittaci* possessed a more stable gene repertoire than those of other extracellular bacteria, with ~90% of the genes per genome being conserved core genes. Furthermore, the evidence for significantly positive selection was identified in 20 virulence-associated gene products, particularly bacterial membrane-embedded proteins and type three secretion machines, which may play important roles in the pathogen-host interactions. This survey uncovered novel strains of *C. psittaci* causing pneumonia and the evolutionary analysis characterized prominent gene candidates involved in bacterial adaptation to immune pressures. The metagenomic approach is of significance to the surveillance of difficult-to-culture intracellular pathogens and the research into molecular epidemiology and evolutionary biology of *C. psittaci*.

## Introduction

*Chlamydia psittaci* is a gram-negative, obligate intracellular pathogen belonging to the family *Chlamydiaceae* (Beeckman and Vanrompay, 2009). It is a zoonotic agent that can cause infections in humans and a broad range of animals, also known as psittacosis (Cong et al., 2013; Vorimore et al., 2015). In people, psittacosis generally presents mild symptoms, including fever, headache, dry cough, and muscle aches; while it can also lead to life-threatening pneumonia, especially in immunocompromised patients (Branley et al., 2016). The agent has been frequently detected in avian and non-avian animals, which has posed economic impacts on the poultry and livestock industry (Knittler and Sachse, 2014). Based on sequencing of the major outer membrane protein OmpA, *C. psittaci* has been divided into nine genotypes, of which genotypes A and E have been described as common etiologic agents causing respiratory illnesses in humans (Van Lent et al., 2012; Wolff et al., 2015).

Clinically, it is difficult to diagnose psittacosis and to detect the related pathogens directly from the specimens through traditional culture, due to *Chlamydia* spp. being strictly intracellular bacteria (Wu et al., 2021). The serological assay and polymerase chain reaction can be utilized to identify the pathogens in patients with suspected psittacosis (Nieuwenhuizen et al., 2018). However, it is impractical to foresee the culprit causing infection in prior in most cases. The application of metagenomic next-generation sequencing (mNGS) has effectively made up for this issue by summarizing the microbial profile in the specimen (Gu et al., 2019). For instance, Chen et al. (2020) have reported that nine cases of pneumonia have negative results for the routine etiological pathogen tests, and these are positive for *C. psittaci* DNA fragments by mNGS. In addition, mNGS enables providing comprehensive genetic information to capture abnormal changes in microbial compositions *in vivo* (Yin et al., 2021). A recent study has revealed that the abundance outliers of certain microbes could prompt their pathogenic risk, and further infer if opportunistic pathogens are harmful or commensal in the niches (Chen et al., 2022).

Decoding genome sequences of the target pathogens from microbiome data is meaningful for tracing microbial evolution and transmission, as well as genetic characteristics of clinically relevant genotypes. Lam et al. (2021) have presented a genomic surveillance framework to detect and type *Klebsiella pneumoniae* and virulence loci genotypes direct from gut metagenome data. Bacterial colonization and transmission patterns in a newly built hospital have been deciphered by metagenomic analysis, which uncovered persistent unique genotypes of *Staphylococcus* and *Propionibacterium* circulating in healthcare environments (Lax et al., 2017). To date, there have been few surveys on the utility of metagenomic surveillance for understanding the genomic dynamics and evolutionary biology of *C. psittaci*.

In this study, *C. psittaci* was detected with significant abundance in the bronchoalveolar lavage fluid (BALF) of two pneumonia human cases. Nearly-complete genomes of *C. psittaci* were reconstructed directly from the shotgun metagenomic sequencing on the specimens. Genomic analyses combined with the public isolates of *C. psittaci* were performed to characterize the molecular epidemiology and pan-genomic structure of the metagenome strains. Furthermore, evidence for positive Darwinian selection acting on virulence-associated genes was first revealed to explore the molecular mechanisms of adaptive evolution for this intracellular pathogen.

## Materials and methods

### Specimen collection

The two patients included in this study had an acute onset of pneumonia. A 72-year-old male patient (#1) with a history of pigeon breeding was admitted to Shanghai General Hospital, Shanghai JiaoTong University School of Medicine (Shanghai, China), in December 2020 due to cough, fever, and dyspnea for 2 weeks. A 66-year-old male patient (#2), a poultry breeder, was admitted to Shanghai Jiao Tong University Affiliated Sixth People’s Hospital (Shanghai, China) in June of 2021 due to fever and chills for 5 days, and chest distress and shortness of breath for 2 days. The patient had a maximum body temperature of 40°C, asthma, decreased appetite, and general fatigue. BALF was harvested in strict accordance with clinical practice. The samples were then sent for mNGS at Genoxor (Shanghai, China). Patient characteristics and clinical tests are given in Supplementary Table S1. Anti-infective therapy with third-generation cephalosporins was started according to the guidelines on the treatment of adult community-acquired pneumonia (Qu and Cao, 2016).

### Metagenomic sequencing and taxonomic classification

Samples were processed through 10,000 *g* centrifugation for 5 min, and the pellets were collected for nucleic acid extraction using DP710-T2A (TIANGEN, China). Approximately 10 ng DNA was used for library construction with the Hieff NGS OnePot Pro DNA Library Prep Kit (Yeasen Biotech., China). Using the mode of single-end 76-bp reads, the library was sequenced by the Illumina NextSeq 550 platform (Illumina Inc., United States). The raw sequencing reads were trimmed and filtered using the program Fastp v0.21.1 (Chen et al., 2018). Host-derived reads were further subtracted by aligning the sequences to the human reference genome GRCh38 using Bowtie v2.2.6 (Langmead and Salzberg, 2012). The remaining reads were used to compute the taxonomic abundances of individual species by Kraken v2.0.9 (Wood et al., 2019) and Bracken v2.2 (Lu et al., 2017).

### Genome assembly of the target pathogen

Bacterial genomes (<200 contigs) of *C. psittaci* isolates were retrieved from the NCBI Assembly database in March 2022 (Kitts et al., 2016; Supplementary Table S2). For the specimens sequenced in this study, the metagenomic reads derived from *C. psittaci* were first extracted based on the reads mapping to the genomes using BBmap v38.18. The species-specific reads were assembled by Spades v3.15.4 with the options -t 24 -m 128 --cov-cutoff auto --isolate (Bankevich et al., 2012). Quality assessment of the resulting assemblies was conducted by both programs QUAST v5.0.2 (Gurevich et al., 2013) and CheckM v1.0.18 (Parks et al., 2015).

### Comparative genome analysis

Average nucleotide identity (ANI) between genomes was calculated by using PYANI v0.2.11 with the option -m ANIb (Pritchard et al., 2016). The resulting matrix was visualized using the R package pheatmap v1.0.12. Multilocus sequence typing (MLST) was performed to detect bacterial sequence types (STs) by Fastmlst v0.0.15 (Guerrero-Araya et al., 2021) according to the scheme *Chlamydiales* with seven alleles: *gatA*, *oppA*, *hflX*, *gidA*, *enoA*, *hemN*, *fumC*. New STs in the genome assemblies generated above were submitted to the PubMLST website (Jolley et al., 2018). For the pan-genome analysis, all the genomes were annotated uniformly by Prokka v1.14.6 (Seemann, 2014). Gene clustering was performed using Roary v3.13.0 (Page et al., 2015) with the options -p 36 -i 80 -e -n -t 11 -s -cd 100 -a -v. A concatenated alignment of core genes was then generated to build a maximum-likelihood phylogenomic tree that was visualized with ggtree v3.2.1 (Yu et al., 2018). Genome-scale sequence conservation was investigated between the metagenome strains and reference strains of nine *C. psittaci* genotypes (Van Lent et al., 2012). A circular genome map was constructed by using BRIG v0.95 (Alikhan et al., 2011).

### Functional analysis of virulence genes

For protein functional classification, we employed Blastp v2.9.0+ (Shiryev et al., 2007) with the *E*-value threshold of 1e^−20^ searching against the COG database. Using the VFDB core database with 4,194 protein sequences (Liu et al., 2018), the genes encoding virulence factors (VFs) were detected by Blastp with the *E*-value threshold of 1e^−20^. Protein subcellular localization was predicted by PSORTb v3.0.3 (Yu et al., 2010). Secondary structure prediction of the proteins was performed using PROTEUS2 (Montgomerie et al., 2008). Protein structure modeling was carried out using the intensive mode of Phyre2 web portal (Kelley et al., 2015). Selection pressure acting on the virulence-associated genes (VAGs) was detected by the PAML v4.9 package (Yang, 2007), which can estimate the ratios (*ω*) of non-synonymous (*d*_N_) to synonymous (*d*_S_) substitutions among the aligned codons. Briefly, protein multiple sequence alignments were initially performed by MAFFT v7.505 (Katoh et al., 2019). Codon alignments were then generated using amino acid sequence alignment and nucleotide sequences per gene. The resulting alignment per gene was further improved to remove the unreliable aligned sites using Gblocks v0.91b (Talavera and Castresana, 2007). The maximum-likelihood algorithm was used to build gene trees by PhyMLv3.1 (Guindon et al., 2010). The codon alignments and the corresponding trees were used for positive selection scanning by the *codeml* program in PAML. Two site-specific models M1a (NearlyNeutral) and M2a (PositiveSelection) were applied herein. Likelihood ratio test (LRT) of positive selection was performed to infer if the data is a significantly better fit for the alternative model M2a, which allows some sites with *ω* > 1; whereas the null model M1a does not allow this. Posterior probabilities for each site were then calculated using Bayes empirical Bayes and the sites with *ω* > 1 and *p* > 95% were predicted to be under positive selection (Yang et al., 2005).

## Results

### Features of pulmonary infection

In this retrospective study, we investigated the metagenomic data of BALF from two cases of pneumonia. The numbers of species present in the lung microbiome were 69 in patient #1 and 48 in patient #2 (Supplementary Table S3). *C. psittaci* was observed to be the most predominant bacteria in the microbial community, whose abundance was 79.4% in patient #1 and 89.8% in patient #2. It implied that *C. psittaci* was the etiological agent closely related to the pulmonary infection in both cases. According to the results of mNGS, the patient #1 was treated with high-flow nasal oxygenation and the antibiotics doxycycline and moxifloxacin. Consequently, the body temperature of the patient gradually reached the normal level, and he was discharged 14 days later. Patient #2 received 16 days of anti-infection treatment with moxifloxacin and minocycline before discharge.

### Metagenome-assembled genomes of *Chlamydia psittaci*

**Figure 1:**
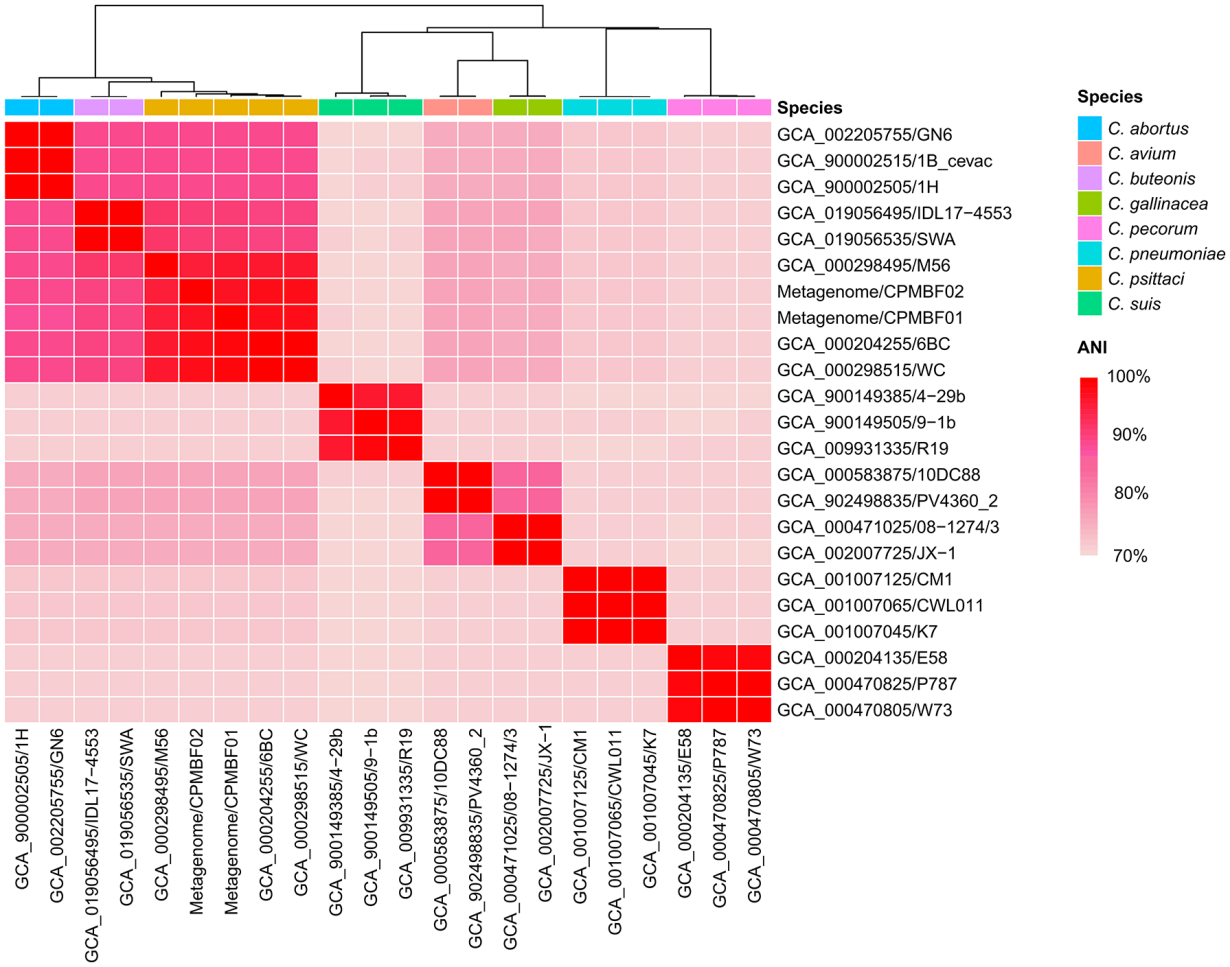
Species identification for the metagenomic strains CPMBF01 and CPMBF02 recovered from the BALF specimen. The heatmap shows the pairwise ANI values between metagenome-assembled genomes and the isolate genomes from eight species within the genus *Chlamydia*. The sequence accessions of the isolate genomes are listed in Supplementary Table S2.

Next, we reconstructed bacterial genomes of two *C. psittaci* strains from the metagenomes of BALF: CPMBF01 and CPMBF02. The assembly metrics are summarized in Table 1. The genome sizes were 1,176,486 bp for CPMBF01 and 1,158,597 bp for CPMBF02, with >99% completeness for both assemblies. The numbers of protein-coding sequences (CDSs) were 978 for CPMBF01 and 975 for CPMBF02. The average GC content of two metagenome-assembled genomes (MAGs) was about 39%, which was consistent with the median GC content of nine *C. psittaci* genotype reference strains (Van Lent et al., 2012). Furthermore, Figure 1 displays the pairwise ANI values between two MAGs and 21 isolate genomes derived from eight species within the genus *Chlamydia*. CPMBF01 shared 97.3% (M56) ~ 99.0% (WC) ANI with three *C. psittaci* isolates (Supplementary Table S4). CPMBF02 shared 97.0% (M56) ~ 98.6% (WC) ANI with three *C. psittaci* isolates. On the other hand, the genomes of CPMBF01 and CPMBF02 possessed relatively low ANI values with the genomes from the other *Chlamydia* species, ranging from 71.3 to 93.5%. The results demonstrate that the strains CPMBF01 and CPMBF02 are derived from *C. psittaci*.

**Table 1:** Genome features of *Chlamydia psittaci* recovered from the metagenomic data of bronchoalveolar lavage fluid in patients with pneumonia.

Strain	CPMBF01	CPMBF02
No. of contigs (>500 bp)	42	25
N50 (bp)	291,009	367,115
NGA50 (bp)	290,877	303,070
Largest (bp)	304,340	472,807
Total (bp)	1,176,486	1,158,597
GC (%)	39.09	39.06
Completeness (%)	99.49	99.49
Contamination (%)	0	0
No. of CDSs	998	989

**Figure 2:**
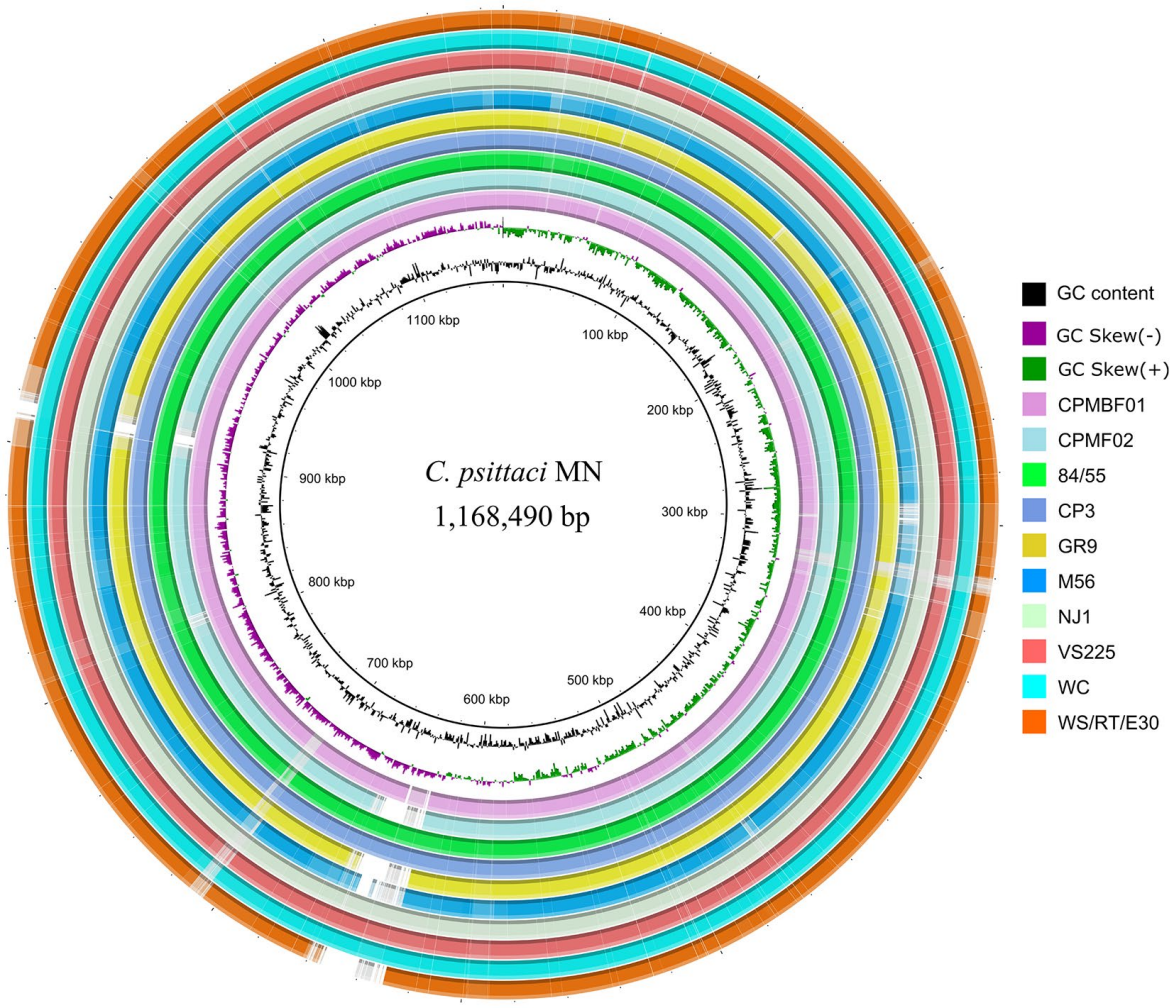
Circular genome representation of eleven *C. psittaci* strains. The innermost two rings denote the tick mark and GC skew plot of the MN genome of *C. psittaci* genotype E. The remaining rings from innermost to outermost represent the two metagenome strains and the isolates from the other eight genotypes, i.e., CPMBF01, CPMBF02, 84/55 of genotype A, CP3 of genotype B, GR9 of genotype C, M56 of genotype M56, NJ1 of genotype D, VS225 of genotype F, WC of genotype WC, and WS/RT/E30 of genotype E/B. The rings corresponding to individual genomes are color-coded based on the metrics of BLAST matches with a minimum sequence identity of 80% and an *E*-value threshold of 1e^−10^.

Furthermore, genome sequence conservation of the metagenomic strains and representative isolates of nine *C. psittaci* genotypes was shown in Figure 2. The highly homologous regions (>95% identity) between the metagenomic strains and genotype E isolate MN accounted for 97.6% (1,140,068 bp; CPMBF01) and 95.0% (1,110,205 bp; CPMBF02) of the MN genome, respectively. Genomic regions (>1 kb) that are present in the MN genome but absent in the MAGs are summarized in Supplementary Table S5. It suggests that the majority of genomic sequences are very conserved across the *C. psittaci* strains.

### Genomic epidemiology of *Chlamydia psittaci*

**Figure 3:**
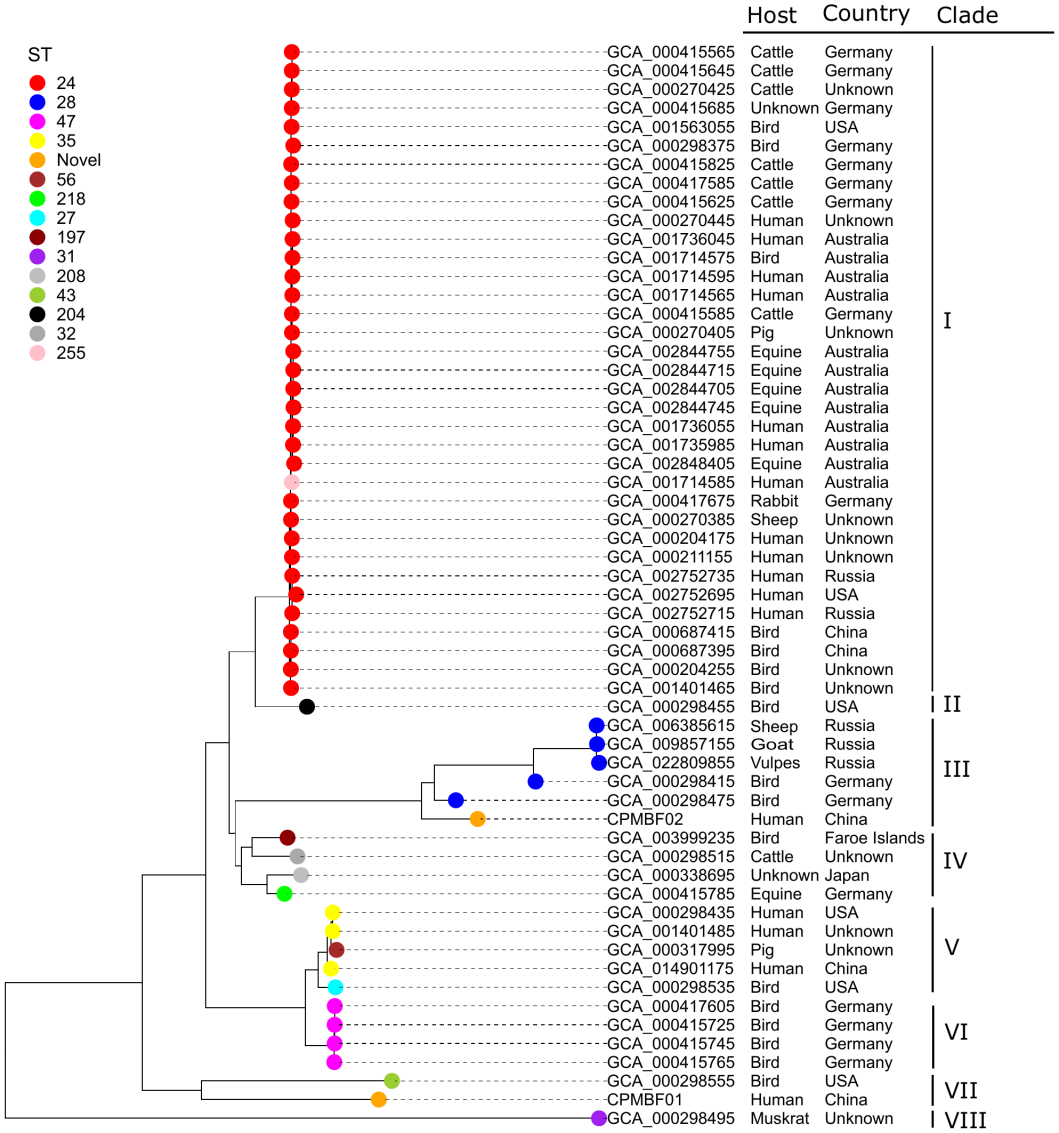
Maximum-likelihood phylogenetic tree of *C. psittaci*. The tip nodes are color-coded by different sequence types. Both metagenomic strains CPMBF01 and CPMBF02 with novel sequence types ST335 and ST334 are represented as orange nodes. The isolation countries, hosts, and evolutionary clades of individual strains are displayed in the right panel.

The relationships between the two metagenomic strains and 56 isolates of *C. psittaci* were analyzed according to a phylogenetic tree built with a core-gene alignment (560,571 nt). Figure 3 shows the strain phylogeny integrated with the bacterial metadata (Supplementary Table S2). Among 14 STs observed in the isolate genomes, ST24 (34 strains) was the most prevalent, followed by ST28 (5), ST47 (4), and ST35 (3). Novel STs were detected in both metagenomic strains, including 85-78-108-112-13-9-78 for CPMBF01 and 12-13-11-13-13-28-12 for CPMBF02. Both ST profiles have been deposited in PubMLST, with the identifiers ST335 assigned to CPMBF01 and ST334 to CPMBF02. As shown in the tree, the strains were distributed into eight clades. In clade VII, the strain CPMBF01 of ST335 was neighboring to the genotype D strain NJ1 (GCA_000298555; ST43), which was isolated from *Meleagris gallapavo*, a large ground-dwelling bird. The strain CPMBF02 of ST334 was clustered with five ST28 strains, two of which were isolated from birds in Germany and the others were from sheep, goats and vulpes in Russia. In terms of geographical distribution, most strains were sampled from Germany (16), Australia (12), the United States (6), China (5), and Russia (5). The frequency of the isolation countries by different hosts is illustrated in Supplementary Figure S1. It seemed that the phylogenetic topology of most strains was consistent with the groups of STs, which was irrelevant to the places of isolation or host preference. For instance, all the strains of ST24 clustered together in clade I were from multiple sources, most of which were isolated from humans (11), birds (7), cattle (7), and equine (5). Of these, the strains of human origin are closely related to the strains from mammals and birds. It indicates that infection caused by *C. psittaci* may be attributed to its wide zoonotic transmission all over the world.

### Pan-genomic composition of *Chlamydia psittaci*

**Figure 4:**
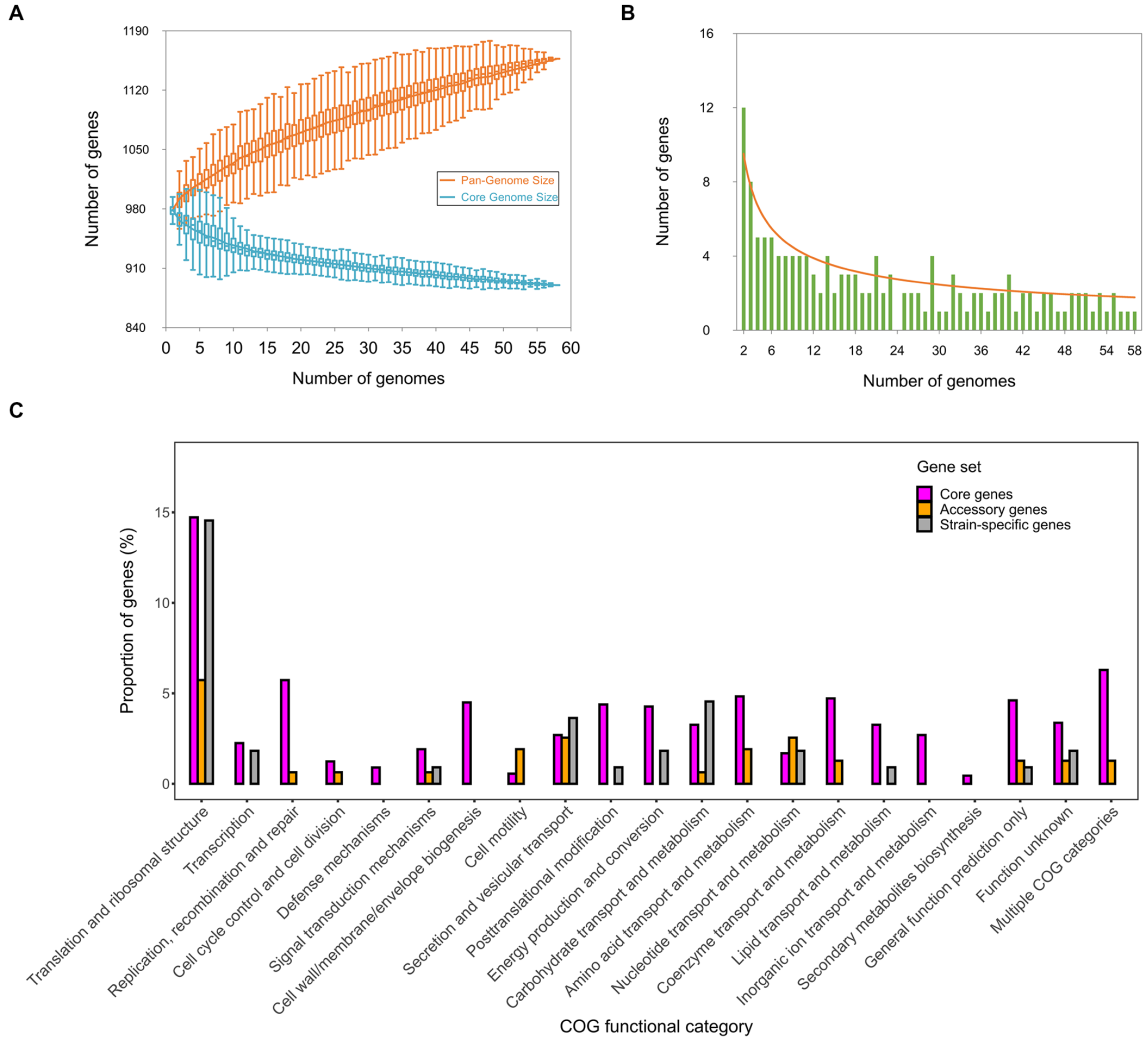
The pan-genomic structure and function of *C. psittaci*. **(A)** Estimation of the sizes of the pan-genome and core-genome is shown by **(A)**, and estimation of the number of strain-specific genes per genome is shown by **(B)**. **(C)** Distribution of the core genes, accessory genes, and strain-specific genes affiliated with individual COG functional categories. The COG assignments of all genes are summarized in Supplementary Table S5.

Based on the clustering of 58,326 CDSs across all the strains, 1,157 orthologous genes (OGs) were detected in the pan-genome of *C. psittaci* (Supplementary Table S6). Of these, over three-quarters (*n* = 890) were conserved core genes present in all the strains. Besides, 157 (14%) accessory genes and 110 (10%) strain-specific genes were found in the pan-genome. Using an exponential decay model on 58 genomes, the number of core genes in *C. psittaci* was estimated to be ~880 genes (Figure 4A), accounting for ~90% of the genes per genome. Notably, there are few strain-specific genes (~3 genes) per newly sequenced *C. psittaci* genome (Figure 4B). In contrast, the gram-negative model organism *Escherichia coli* encodes about 300 truly unique genes per genome and the percentage of the core genes per genome is ~47% (Rasko et al., 2008). It was apparent that *C. psittaci* possessed a stable gene repertoire and a weakening ability in the acquisition of genetic materials, perhaps due to the strictly intracellular properties of this bacterium. In addition, the proportion of three gene-sets distributed in the COG functional categories is shown in Figure 4C. Except for the genes encoding unknown functions, the most abundant category is “Translation and ribosomal structure,” with 131 genes. Several categories consisted of conserved core genes without the other dispensable genes, such as “Cell wall/membrane/envelope biogenesis,” “Defense mechanisms,” and “Inorganic ion transport and metabolism.” Generally, the genic components of *C. psittaci* tend to be more conserved than other extracellular bacterial pathogens whose horizontal gene transfers are vibrant (Arnold et al., 2022).

### Positive selection on virulence genes of *Chlamydia psittaci*

To depict genetic features of virulence factors possessed by *C. psittaci*, 168 VAGs were identified in the bacterial pan-genome. The majority of VAGs (*n* = 158) were core genes (Supplementary Table S5). Based on the classification of virulence factors, the VAGs were assigned to 12 functional groups. The largest group “Effector delivery system” included 85 genes, followed by “Adherence” with 23 genes, “Immune modulation” with 20 genes, “Nutritional/metabolic factor” with 15 genes, and “Stress survival” with eight genes. A full spectrum of genes coding for products from classical virulence factors in *Chlamydia* was identified. For instance, we detected 30 genes involved in the assembly of type III secretion system (T3SS) and 49 genes encoding effector proteins secreted by T3SS (Supplementary Table S6). A total of 16 genes encoding polymorphic membrane proteins (Pmps), which can be vital for mediating adhesion to host cells (Favaroni and Hegemann, 2021), were detected in the pan-genome of *C. psittaci*.

**Figure 5:**
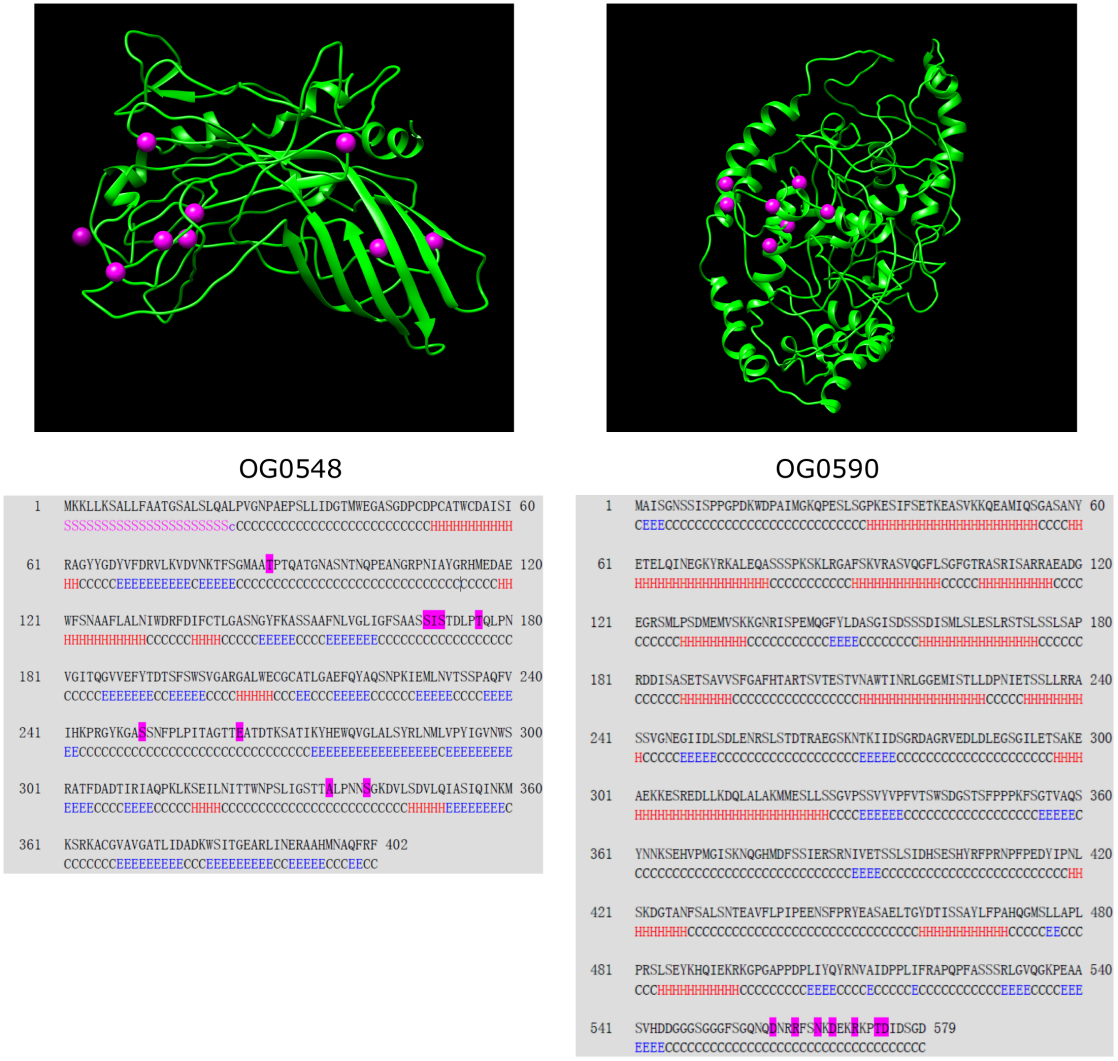
Secondary and tertiary structure of the outer membrane proteins encoded by two *C. psittaci* genes. Positively selected sites are illustrated by magenta spheres in the structural models (upper panel) and magenta residues in the corresponding sequences (lower panel). The segments of the secondary structure are marked by ‘H’ for helix, ‘E’ for β-strand, ‘C’ for coil, ‘S’ for signal peptide, and ‘c’ for cleavage site. Both proteins are identical to the following NCBI reference sequences: OG0548 to WP_006342723 (402 aa) and OG0590 to WP_006343129 (579 aa). The protein sequences displayed herein are provided in Supplementary Data Sheet 1.

Next, we investigated the evidence for positive selection operating on the 135 single-copy core genes associated with bacterial virulence and cell surface components. According to LRT between the null model M1a and the selection model M2a, 20 *C. psittaci* genes were identified as undergoing strong positive selection (*p*-value < 0.05; Table 2 and Supplementary Table S7). Half of these genes were encoding membrane-associated proteins whose adaptive evolution might be actively induced by selective immune pressure. For instance, the products encoded by both genes OG0548/0590 were predicted to localize on the bacterial outer membrane. Figure 5 shows structural models of the outer membrane proteins and the relevant sites subject to positive selection.

**Table 2:** Positively selected genes encoding proteins involved in the cell wall biogenesis and virulence of *C. psittaci*.

OG ID	Symbol	*p* ^a^	*ω*	2Δln*L*^b^	Annotation	COG^c^	Localization^d^
OG0373	*aaxA*	0.002	76.914	15.836	Porin AaxA	M	NA
OG0425	*mrcA*	0.055	82.707	30.740	hypothetical protein	–	CM
OG0448	*murG*	0.055	30.765	25.658	UDP-N-acetylglucosamine--N-acetylmuramyl-(pentapeptide) pyrophosphoryl-undecaprenol N-acetylglucosamine Transferase	M	CM
OG0468	*–*	0.083	3.304	8.298	Hypothetical protein	M	CM
OG0482	*CT274*	0.050	96.681	45.498	Hypothetical protein	R	CM
OG0498	*copD2*	0.024	9.312	8.347	Hypothetical protein	–	CP
OG0502	*murB*	0.039	19.631	9.210	UDP-N-acetylenolpyruvoylglucosamine reductase	M	NA
OG0536	*tlyC*	0.164	3.491	26.544	Hypothetical protein	R	CM
OG0548	*ompA*	0.070	4.309	46.986	Major outer membrane porin	–	OM
OG0549	*copD*	0.209	2.165	10.242	Hypothetical protein	–	NA
OG0561	*CT_203*	0.064	13.160	22.323	Hypothetical protein	S	CP
OG0569	*yycJ*	0.089	201.954	77.733	Putative metallo-hydrolase YycJ	P	CP
OG0586	*–*	0.033	22.052	10.168	Hypothetical protein	MO	NA
OG0590	*CT_082*	0.032	15.318	26.467	Hypothetical protein	–	OM
OG0612	*mcsc*	0.022	13.642	6.931	Hypothetical protein	S	CP
OG0722	*cdsZ*	0.085	7.078	23.544	Hypothetical protein	R	CP
OG0737	*epsE*	0.058	29.561	69.307	Type II secretion system protein E	NUW	CP
OG0765	*cdsG*	0.055	13.051	14.970	Hypothetical protein	R	NA
OG0774	*NUE*	0.078	9.692	15.289	hypothetical protein	R	CP
OG0781	*fliI*	0.048	9.136	20.913	Flagellum-specific ATP synthase	NU	CP

The percentage of positively selected amino acid residues.

## Discussion

The incidences of lung infection caused by *C. psittaci* are usually underestimated due to complicated clinical presentations varying from severe pneumonia to asymptomatic infection (De Gier et al., 2018). Diagnosis of psittacosis can be challenging through routine methods for detecting the infectious agent *C. psittaci* in the hospital setting (Nieuwenhuizen et al., 2018). Like *C. trachomatis*, *C. psittaci* also lacks genetic manipulation systems to resolve the function of genes (Kari et al., 2011). To better depict the molecular features of this difficult-to-culture intracellular pathogen, we reconstructed the draft genomes of *C. psittaci*, and further characterize the phylogeny of novel strains, pan-genomic structure and adaptive evolution of virulence genes under a metagenomic framework.

The obligate intracellular lifestyle of *Chlamydia* spp. may restrict genic acquisition via lateral transfer in the bacterial community (Stepkowski and Legocki, 2001). It was worth noting that the genes encoding structural proteins responsible for the biosynthesis of T4SS were absent in the pan-genome of *C. psittaci* (Supplementary Table S6). Meanwhile, *C. psittaci* possesses a high proportion (~90%) of core genes per genome, which is similar to that (~92%) of *C. trachomatis* (McInerney et al., 2017). Based on the metric, *Chlamydia* spp. have relatively low genomic plasticity in comparison to other intracellular bacteria, like *Anaplasma phagocytophilum* (~72%) and *Coxiella burnetii* (~60%) (Dugat et al., 2014; Abou Abdallah et al., 2022). In contrast, the proportion of conserved core genes is lower in the extracellular bacteria, such as *E. coli* (~47%), *Pseudomonas aeruginosa* (~41%), and *Klebsiella pneumoniae* (~32%) (Mosquera-Rendón et al., 2016; Wyres et al., 2020). It implies that natural selection or other forces driving the evolution of *Chlamydia* spp. may be reflected by small genetic variations in the coding regions rather than frequent gene gain or loss.

Chlamydial virulence factors, particularly surface appendages, play key roles in the interaction with the host immune system and are associated with prevalence and disease pathogenesis (Byrne, 2010). Herein, we focused on 20 *C. psittaci* VAGs showing a significant signal of positive selection. Half of these genes code for products localized on the cell surface/membrane, which are prone to natural selection pressures and other evolutionary forces (Xu et al., 2011). For instance, OmpA encoded by OG0548 (402 aa) shares 64.9% amino acid sequence identity with the homolog MOMP (393 aa) in *C. trachomatis* D/UW-3/CX, which acts as a cytoadhesin involved in the binding of chlamydial elementary bodies to heparan sulfate proteoglycans on the host cells (Su et al., 1996). Nine positively selected amino acid residues were detected in *C. psittaci* OmpA (Figure 5), the genetic changes of which may be associated with the diversity of bacterial serotypes (Knittler and Sachse, 2014). Another positively selected gene OG0373 (448 aa) encodes a putative porin belonging to the OprB family (PF04966) of carbohydrate-selective porins and shares 75% similarity with an integral outer membrane protein AaxA (438 aa, WP_014518085) in *C. pneumoniae*, which can enhance arginine uptake and decarboxylation activity of bacterial cells (Smith and Graham, 2008).

Besides, the roles of the effector delivery system in chlamydial infection have been extensively studied, such as attachment and invasion into host cells, regulation of intracellular trafficking and innate immune signaling (Mojica et al., 2015; Carpenter et al., 2017; Sixt et al., 2017). In *C. psittaci*, we detected 13 positively selected genes involved in the effector delivery system, including five genes of T3SS, one gene of T2SS, and seven effector genes (Supplementary Table S7). Of these, both genes OG0549/0498 (440 aa/494 aa) encoding translocators are homologous to the *C. pneumoniae* CopD (444 aa) and CopD2 (497 aa), of which CopD has been verified as a hydrophobic translocator with functions in bacterial infectivity (Bulir et al., 2014). The product of OG0590 (579 aa), another positively selected outer membrane protein of *C. psittaci* (Figure 5), shares 44.9% identity with *C. trachomatis* CT_082 (560 aa) coding for a T3SS secreted effector. Additionally, significant evidence for positive selection on *yycJ* (262 aa) coding for a metallo-hydrolase was identified (*p* < 0.001; Supplementary Table S7), which shares 55% similarity with a zinc metallo-beta-lactamase (264 aa) from *Bacillus cereus* (Carfi et al., 1995). YycJ is a pleiotropic protein with a domain (Lactamase_B, PF00753) catalyzing the hydrolysis of most beta-lactams that may facilitate enzymatic evolution for bacterial survival (Bebrone, 2007). Since genetic intractability for *Chlamydia* spp., decoding the function of their genes has remained challenging (McClure et al., 2017). Detection of genes subject to positive selection should provide evidence-based candidates for elucidating the molecular mechanisms of adaptation and immune evasion for *C. psittaci*.

In this study, we performed comparative genomic analysis of 56 *C. psittaci* isolates and two novel strains directly from the BALF specimens of pneumonia. New sequence types ST335 and ST334 were assigned to the two metagenomic strains that were closely related to the animal-borne isolates belonging to ST43 and ST28. The pan-genome analysis revealed this obligate intracellular pathogen exhibited a low genomic plasticity represented by a highly conserved core-genome. Furthermore, we identified 20 positively selected virulence genes and related amino acid sites showing evidence for adaptive evolution and pathogen-host interactions. Our findings indicate the metagenomic technique will be a new approach to clinical surveillance and research into the epidemiology and evolutionary biology of difficult-to-culture pathogens.

## Data availability statement

The datasets presented in this study can be found in online repositories. The names of the repository/repositories and accession number(s) can be found at: https://www.ncbi.nlm.nih.gov/, PRJNA895174.

## Ethics statement

The studies involving human participants were reviewed and approved by the Ethics Committee of Shanghai Sixth People’s Hospital. The patients/participants provided their written informed consent to participate in this study.

## Author contributions

YL, YF, and RW conceived and designed the study. WH, SH, and XZ performed the experiments. WH, SH, YL, and RW contributed to materials. WH, YF, YL, SL, and YZ performed sequence analysis and data interpretation. WH, SH, YZ, and XZ wrote the manuscript. SL, YL, YF, and RW provided major revisions to the manuscript. All authors contributed to the article and approved the submitted version.

## Funding

This work was supported by the Shanghai Municipal Science and Technology Major Project (grant no. ZD2021CY001).

## Conflict of interest

XZ and YF were employed by Genoxor Medical Science and Technology Inc.

The remaining authors declare that the research was conducted in the absence of any commercial or financial relationships that could be construed as a potential conflict of interest.

## Publisher’s note

All claims expressed in this article are solely those of the authors and do not necessarily represent those of their affiliated organizations, or those of the publisher, the editors and the reviewers. Any product that may be evaluated in this article, or claim that may be made by its manufacturer, is not guaranteed or endorsed by the publisher.
