# Supplementary material for: Metagenomic surveillance and comparative genomic analysis of Chlamydia psittaci in patients with pneumonia
Source: Front Microbiol. 2023 May 30;14:1157888. doi: 10.3389/fmicb.2023.1157888 (PMC10265514; doi:10.3389/fmicb.2023.1157888)
Supplement: Supplementary file 9 [file Image_1.pdf]

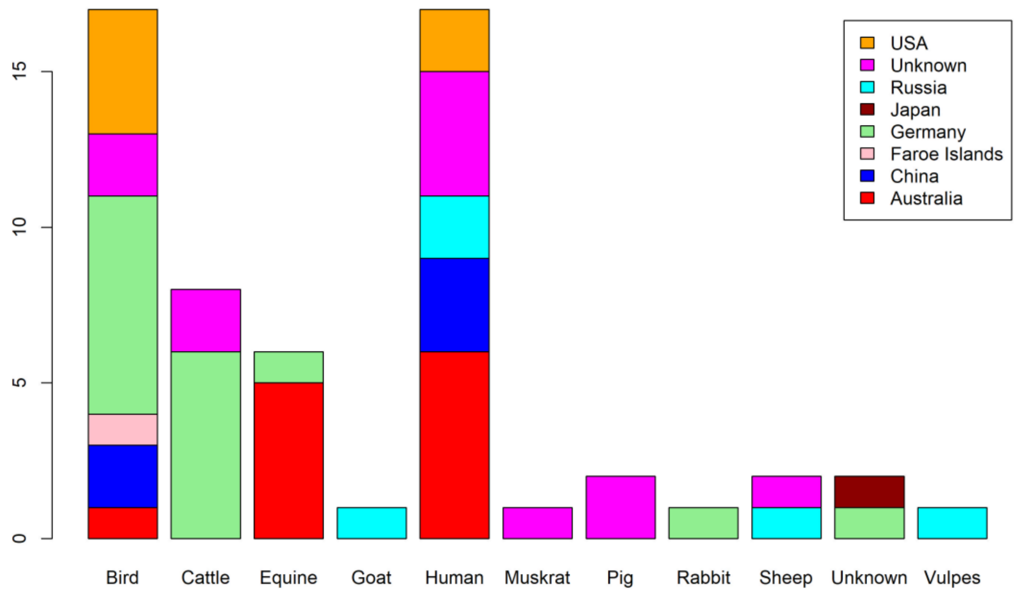

Figure S1. The stacked barplot showing the frequency of the isolation countries by different hosts.
